# Exploring spatial variations and factors associated with childhood stunting in Ethiopia: spatial and multilevel analysis

**DOI:** 10.1186/s12887-016-0587-9

**Published:** 2016-04-15

**Authors:** Demewoz Haile, Muluken Azage, Tegegn Mola, Rochelle Rainey

**Affiliations:** 1 Department of Public Health, College of Medicine and Health Sciences, Bahir Dar University, P.O. Box 79, Bahir Dar, Ethiopia; 2 Department of Geography and Environmental Studies, Bahir Dar University, P.O. Box 79, Bahir Dar, Ethiopia; 3 United States Agency for International Development (USAID), Washington, DC USA

**Keywords:** Stunting, Children, Ethiopia

## Abstract

**Background:**

Stunting reflects a failure to receive adequate nutrition over a long period of time. Stunting is associated with adverse functional consequences including poor cognition, low educational performance, low adult wages, and poor reproductive outcomes. The objective of the study was to investigate spatial variations and factors associated with childhood stunting in Ethiopia.

**Methods:**

This study is a secondary data analysis of the 2011 Ethiopian Demographic and Health Survey (EDHS). A total of 9893 children aged 0–59 months were included in the analysis. The Getis-Ord spatial statistical tool was used to identify high and low hotspots areas of stunting. A multilevel multivariable logistic regression was used to identify factors associated with stunting.

**Results:**

Statistically significant hotspots of stunting were found in northern parts of the country whereas low hotspots where there was less stunting than expected were found in the central, eastern, and western parts of the country. In the final model of multilevel logistic regression analysis, individual and community level factors accounted for 36.6 % of childhood stunting. Short birth interval [AOR = 1.68; 95%CI: (1.46–1.93)], being male [AOR = 1.20; 95%CI: (1.08–1.33)], and being from a male-headed household [AOR = 1.18; 95 % CI: (1.01–1.38)] were the factors that increased the odds of stunting at the individual level. Children in the age group between 24–35 months were more likely to be stunted than children whose age was less than one year [AOR = 6.61; 95 % CI: (5.17–8.44)]. The odds of stunting among children with severe anemia were higher than children with no anemia [AOR = 3.23; 95%CI: (2.35–4.43)]. Children with mothers who had completed higher education had lower odds of being stunted compared to children whose mothers had no formal education [AOR = 0.42; 95%CI: (0.18–0.94)]. The odds of being stunted were lower among children whose fathers completed higher education [AOR = 0.58; 95%CI: (0.38–0.89)] compared to children whose fathers had no formal education. Children whose mothers who had high a Body Mass Index (BMI) (≥25.0 kg/m^2^) were less likely to be stunted compared with children whose mothers had a normal BMI (18.5 kg/m^2^-24.9 kg/m^2^)[AOR = 0.69; 95%CI: (0.52–0.90)]. Children from the poorest wealth quintile had higher odds of being stunted compared to children from the richest wealth quintiles [AOR = 1.43; 95 % CI: (1.08–1.88)]. Unavailability of improved latrine facilities and living in the northern parts of the country (Tigray, Affar, Amhara and Benishangul-Gumuzregions) were factors associated with higher odds of stunting from the community-level factors.

**Conclusion:**

Stunting in children under five years old is not random in Ethiopia, with hotspots of higher stunting in the northern part of Ethiopia. Both individual and community-level factors were significant determinants of childhood stunting. The regions with high hotspots of child stunting should be targeted with additional resources, and the identified factors should be considered for nutritional interventions.

## Background

Height-for-age Z-score is used as an indicator of linear growth retardation and cumulative growth deficits in children [1]. Stunting is defined as a child with a height-for-age Z-score (HAZ) less than minus two standard deviations (<−2 SD) below the median of a reference height-for-age standard. Stunting reflects a failure to receive adequate nutrition over a long period of time, and is affected by both recurrent and chronic illness [2]. Stunting in childhood is associated with adverse functional consequences later in life including poor cognition, poor educational performance, low adult wages and poor reproductive outcomes [3, 4]. Stunted children also have a higher risk of being overweight or obese later in life, putting them at risk of chronic disease in adulthood [3, 5–7].

Childhood stunting varies not only across various regions of the world but also within and between local authorities, regional space dimensions and/or countries [8]. Although Ethiopia has made steady progress in reducing stunting (from 2000–2011 stunting declined from 58 % to 44 %)[9, 10], the prevalence of stunting is still one of the highest in the world [10], and the issue is a national priority. In order to accelerate efforts in reducing stunting, along with other nutritional problems, Ethiopia revised its national nutrition program in 2013 [11]. Identifying hotspots, or areas where the prevalence of stunting is higher than the national average, would help the Ethiopian Government strategically intensify interventions in order to reduce the prevalenceof stunting in the country.

There aremany studies which determine the prevalence of stunting and analyze socioeconomic, demographic and cultural factors associated with childhood stunting in Ethiopia [12, 13]. However, no spatial analysis has identified the hotspotsof stunting in the country. Moreover, many of the previous studies used standard logistic regression to identify the independent predictors of stunting. Analyzing variables from different levels at one single common level using the standard binary logistic regression model leads to biased results (loss of power or Type I error). Households in the same geographic cluster have common characteristics such as seasonal variability, types of crops and housing which can have a similar impact on the nutritional status of children in the cluster. The assumptions of independence among individuals within the same cluster and of equal variance across clusters are violated in the case of grouped data. Hence, a multilevel analysis is the appropriate statistical analysis method for such a study. This study employed a multilevel logistic regression analysis which has a number of advantages over standard logistic regression, as described in detail by Guo and Zhao [14]. This study aimed to investigate the spatial variation of stunting and the factors associated with stunting in Ethiopia using spatial and multilevel analyses.

## Methods

### Study design and setting

An in-depth secondary data analysis was conducted using Ethiopian Demographic and Health Survey (EDHS) data from 2011. The EDHS is carried out every five years to provide health and health-related indicators at the national and regional levels in Ethiopia. Administratively, regions in Ethiopia are divided into zones, and zones, into administrative units called woredas. Each woreda is further subdivided into the lowest administrative unit, called kebeles. During the 2007 census each kebele was subdivided into census enumeration areas (EAs), which were convenient for the implementation of the census. The 2011 EDHS sample was selected using a stratified, two-stage cluster design where EAs were the sampling units for the first stage, and households for the second stage. The detailed sampling procedure is presented in the full EDHS report [10].

### Measurement

The length of children aged < 24 months was measured during the EDHS in a recumbent position to the nearest 0.1 cm using a locally made board with an upright wooden base and movable headpieces. Children ≥24 months were measured while standing upright. The height-for-age Z-score, an indicator of nutritional status, was compared with reference data from the WHO Multicentre Growth Reference Study Group, 2006 [15]. Children whose height-for-age Z-score is < −2 SD from the median of the WHO reference population are considered stunted (short for their age). A wealth index was constructed using principal components analysis on household asset data to categorize individuals into five wealth quintiles (poorest, poorer, medium, richer and richest). Variables included in the wealth index were ownership of selected household assets, size of agricultural land, quantity of livestock and materials used for house construction [16]. Three steps were used in the construction of the wealth index to permit greater adaptability of the wealth index to both urban and rural areas. In the first step, a subset of indicators common to urban and rural areas was used to create wealth scores for households in both areas. In the second step, separate factor scores were produced for households in urban and rural areas using area-specific indicators. The third step combined the separate area-specific factor scores to produce a nationally-applicable combined wealth index by adjusting area-specific scores through a regression on the common factor scores. A more detailed description of the wealth index is presented in the full EDHS report [10].

### Explanatory variables

The individual- and community-level variables included in the study as explanatory variables are shown in Table 1, along with the coding and definitions. Individual-level variables include socio-demographic and economic characteristics (Level one). Community-level variables describe the cluster of the communities living in the same geographical living environment (Level two). These two hierarchal levels were used to create a multilevel analysis for this study. Communities were based on sharing a common primary sampling unit (cluster) within the EDHS data. A multilevel logistic regression model was applied for three reasons. First, in the EDHS sample, primary sampling unit (PSU) was used to define the clusters. Second, it has been shown that for most of the DHS data, the sample size per cluster meets the optimum size with a tolerable precision loss to do a multilevel analysis [17]. Third, multilevel modeling systematically analyzes how covariates at various levels of hierarchal structure affect the outcome variable and how the interactions among covariates measured at different levels affect the outcome variable. Moreover, multilevel modeling corrects for the biases in parameter estimates resulting from clustering and provides correct standard errors [14].

**Table 1:** Variables definition

Individual-level factors	
Child factors	
Age of child (months)	Categorized into (1) 0–11; (2) 12–23; (3) 24–35; (4) 36–47; and (5) 48–59.
Sex of child	Categorized into (1) female and (2) male.
Birth weight (g)	Categorized into (1) low < 2500 and (2) normal ≥ 2500.
Type of birth	Categorized into (1) single and (2) multiple birth
Immunization	Categorized into (1) incomplete or (2) complete
Anemia	Categorized into (1) non-anemic; (2) mild; (3) moderate; (4) sever
Maternal/household factors	
Maternal age in years	Categorized into (1) 15–24; (2) 25–34; or (3) 35–49.
Educational level of mother	Categorized into (1) no formal education; (2) primary; (3) secondary; or (4) higher.
Mother’s body mass index (kg/m2)	Categorized into (1) <18.5; (2) 18.5–24.9; or (3) ≥ 25.0.
Birth interval (months)	Categorized into (1) ≥24 and (2) <24.
Number of under-fives children	Categorized into (1) 1; (2) 2; (3) 3; or (4) ≥4.
Head of household	Categorized into (1) male or (2) female.
Wealth index	Categorized into (1) (first quintile) (Poorest); (2) (second quintile); (3) (third quintile); (4) (fourth quintile); or (5) (fifth quintile) (Richest)
Community-level factors	
Residence Poverty rate	Categorized into (1) rural or (2) urban. Proportion of households living below poverty level (wealth index below 20 %, poorest quintile). Categorized into (1) Low or (2) High. Median value serves as the reference for the low and high groups.
Region	Categorized into (1) Dire Dawa; (2) Tigray; (3) Afar; (4) Amhara; (5) Oromiya; (6)Somali; (7) Benishangul-Gumuz; (8) SNNP; (9) Gambela; (10) Harari; (11) Addis Ababa
Latrine facility type	Categorized into (1) improved (access to flush toilet, ventilated improved pit latrine, traditional pit latrine with a slab, or composting toilet and does not share this facility with other households) or (2) unimproved.
Drinking water sources	Categorized into (1) piped water; (2) Other improved (protected spring and well, and rain water); (3) unimproved (river, pond, unprotected spring and well).

#### Data analysis

Data analysis was carried out using STATA version 12(StataCorp, College Station, Texas, United States) statistical software and spatial analysis was done using ArcGIS software, version 10.0 (ESRI, Redlands, CA, USA). The authors used the “svy” command in STATA version 12 to weight the survey data as per recommendation of the EDHS. Sample weights were applied in order to compensate for the unequal probability of selection between the strata that were geographically defined, as well as for non-responses. A detailed explanation of the weighting procedure can be found in the EDHS methodology report [10]. Multilevel logistic regression was carried out using STATA version 12data analysis and statistical software.

#### Spatial analysis

Spatial analysis was done using GIS Getis-Ord statistics. The prevalence rates of stunting were exported into ArcGIS to visualize key estimations, and the excess risk of stunting of each region was calculated. Excess risk is defined as a ratio of the observed number over the expected number of cases. In this study, the Local Getis-Ord G index (LGi) was applied to do spatial statistical analysis. The LGi is described in detail in the literature [18]. The spatial heterogeneity of significant high prevalence/low prevalence areas of stunting were computed for each cluster using the Getis-Ord G-statistic tools in ArcGIS. The Local Getis-Ord G index helped to classify the autocorrelations into positive and negative correlations. If prevalence rates had similar high values or low values, they were defined as positive autocorrelation hotspots (represented as High-High or Low-Low autocorrelation). If the attributes held opposing high and low values, they were considered to have negative autocorrelation (represent as High-Low or Low-High autocorrelation). To determine the significance of these statistics, Z-scores and P-values were used. A Z-score near zero indicates no apparent clustering within the study area. A positive Z-score with P-value of <0.05 indicates statistical clustering of hotspots of childhood stunting whereas a negative Z-score with p-value of <0.05 indicates statistical clustering of children who are not stunted.

#### Multilevel logistic regression

Multivariable multilevel logistic regression was used to analyze factors associated with childhood stunting at two levels: individual and community (cluster) levels. Four models were constructed for this multilevel logistic regression analysis. The first model was an empty model without any explanatory variables, to evaluate the extent of the cluster variation on stunting. The second model adjusted for the individual-level variables, the third model adjusted for community-level variables while the fourth model adjusted for both the individual- and community-level variables simultaneously. A P-value of <0.05 was used to define statistical significance. Adjusted Odds Ratios (AOR) with their corresponding 95 % confidence intervals (CIs) were calculated to identify the independent predictors of stunting. Intra-cluster correlation (ICC), Median Odds Ratio (MOR) and proportional change in variance (PCV) statistics were calculated to measure the variation between clusters. ICC was used to explain cluster variation while MOR is a measure of unexplained cluster heterogeneity [19]. ICC is the measure of variation attributed to contextual neighborhood factors(residential level factors), and is often used to operationalise the concept of contextual phenomena [20]. The ICC was calculated using this formula [$ICC=\frac{\uptau^2}{\left({\uptau}^2+\frac{\uppi^2}{3}\right)},$ where τ^2^ is the estimated variance of clusters)] described elsewhere [21]. MOR is defined as the median value of the odds ratio between the area at highest risk and the area at lowest risk when randomly picking out two areas and it was calculated using the formula $\left[\mathrm{MOR}= \exp \kern0.28em \left(\sqrt{2\mathrm{x}{\uptau}^2\times 0.6745}\right)\approx \exp \kern0.28em \left(0.95\uptau \right)\right].$ In this study MOR shows the extent to which the individual probability of being stunted is determined by residential area [21]. The proportional change in variance (PCV) measures the total variation attributed by individual level factors and area level factors in the multilevel model. MOR and the formula for PCV have been described elsewhere [20, 22, 23].

### Ethical considerations

The data were downloaded and analyzed after the purpose of the analysis was communicated and approved by MEASURE DHS. The original EDHS data were collected in conformation with international and national ethical guidelines. Ethical clearance for the survey was provided by the Ethiopian Public Health Institute (EPHI), former Ethiopian Health and Nutrition Research Institute (EHNRI) Review Board, the National Research Ethics Review Committee (NRERC) at the Ministry of Science and Technology, the Institutional Review Board of ICF Macro International, and the United States Center for Disease Control and Prevention(CDC).

## Results

### Characteristics of the study participants

Majority of the study subjects (87.1 %) were from rural area. Most of the mothers (69.3 %) had no formal education while 50.22 % of their partners had also no formal education. The poorest wealth quintile comprises about 22.8 % of the total population. A total of 11.43 % of the children were infants less than a year. Most of the children had a preceding birth interval of 24 months and above. Only 7.46 % of the households have improved latrine while 10.36 % used piped water as source of drinking water (Table 2).

**Table 2:** Socio-demographic and economic characteristics of respondents included in the analysis, 2011 EDHS

Variables	Weighted Frequency	Weighted Percent
Place of residence		
Urban	1,528	12.9
Rural	10,344.	87.1
Maternal education		
No formal education	8,227	69.3
Primary	3,211	27.0
Secondary	266	2.2
Higher	168	1.4
Father’s education level		
No education	5,878	50.22
Primary	4,866	41.58
Secondary	584	4.99
Higher	376	3.22
Wealth index		
Richest	2,173	15.1
Richer	1,870	19.1
Middle	1,872	20.5
Poorer	2,114	22.4
Poorest	3,625	22.8
Child’s age(months)		
0–11	1,060	11.43
12–23	1,833	19.76
24–35	1,963	21.17
36–47	2,264	24.41
48–59	2,154	23.23
Sex		
Female	5,704	48.05
Male	6,168	51.95
Anemia		
Non-anemic	4,983	55.40
Mild	1,953	21.71
Moderate	1,839	20.44
Severe	220	2.45
Mother’s BMI (kg/m^2^)		
18.5–24.9	8,605	72.61
<18.5	2,470	20.84
> = 25.0	776	6.55
Birth interval		
<24 months	2,275	23.73
> = 24 months	7,313	76.27
Head of household		
Female	1,767	14.88
Male	10,105	85.12
Region		
Dire Dawa	39	0.3
Tigray	753	6.3
Affar	121	1.0
Amhara	2,656	22.4
Oromiya	5,014	42.2
Somali	364	3.1
Benishangul-Gumuz	140	1.2
SNNP	2,494	21.0
Gambela	40	0.3
Harari	28	0.2
Addis Ababa	222	1.9
Improved latrine facility		
Yes	860	7.46
No	10,668	92.54
Drinking water supply		
Piped water	616	10.36
Other improved	1,084	18.24
Unimproved	4,243	71.39

### Spatial variation of stunting

The overall prevalence of childhood stunting in Ethiopia was 44.5 % (95 % CI: 43.6–45.5). There were regional variations, with Tigray, Amhara, Afar and Benishangul-Gumuzregions having a higher prevalence of stunting, while the lowest prevalence of stunting was found in Addis Ababa(Table 3). Rural areas had a higher prevalence of stunting than urban areas. The most excess cases of childhood stunting were found in Amhara, Tigray and Afar regions, while lower excess risk was found in Addis Ababa and Gambella Regions (Table 3).

**Table 3:** Regional variation of prevalence rate and risk of stunting in Ethiopia, DHS 2011

Region	Weighted number		Weighted Prevalence of stunting (95 % CI)	Excess Risk
	Stunted	Normal		
Tigray	346.	332	51.0(47.3–54.8)	1.15
Afar	50	52	51.0(41.3–60.6)	1.15
Amhara	1175	1,088	51.9(49.9–54.0)	1.17
Oromiya	1,838	2,594	41.5(40.0–43.0)	0.93
Somali	92	189	32.7(27.5–38.5)	0.73
Benishangul-Gumuz	58	60	49.4(40.2–58.1)	1.11
SNNP^a^	965	1204	44.5(42.4–46.6)	1.0
Gambella	9	23	28.0(14.7–45.4)	0.63
Harari	7	16	30.3(14.4–51.1)	0.68
Addis Ababa	39	144	21.7(15.8–27.6)	0.48
Dire Dawa	12	22	36.4(21.4–53.6)	0.79
Place of Residence				
Urban	391	876	30.8(28.4–33.5)	0.69
Rural	4,202	4,844	46.4(45.4–47.5)	1.04
Total	4,593	5721	44.5(43.6–45.5)	

^a^South Nation, Nationalities and region people

**Fig. 1:**
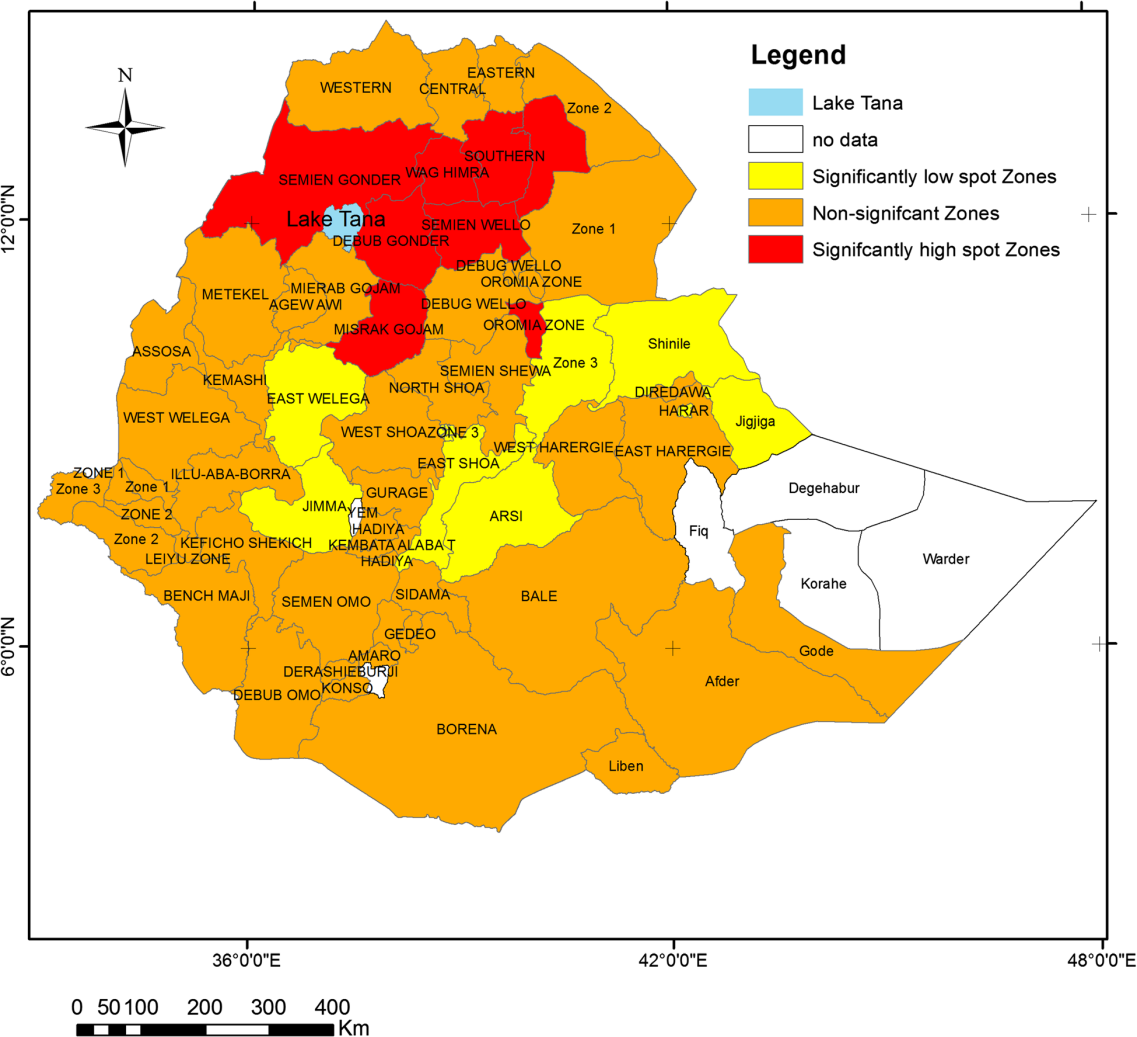
Statistical significant hotspots of childhood stunting at zonal level, DHS 2011

All zones in Addis Ababa, in Harari, four zones in Oromia region (Jimma, East Wollega, Arsi and West Showa) and two zones in Somali region (Jijiga and Shinile) were significantly clustered with low prevalence of stunting (negative Z-score and Gipvalue < 0.05). Five zones in Amhara region (Kamissie zone, Debub Gonder,SemienWello,Wag Himra and Misrak Gojjam), and Southern zone in Tigray region were significantly clustered with high prevalence of stunting (Positive Z-score and Gipvalue < 0.05). The rest of the zones were not significantly clustered with either low or high prevalence of stunting (Fig. 1).

**Fig. 2:**
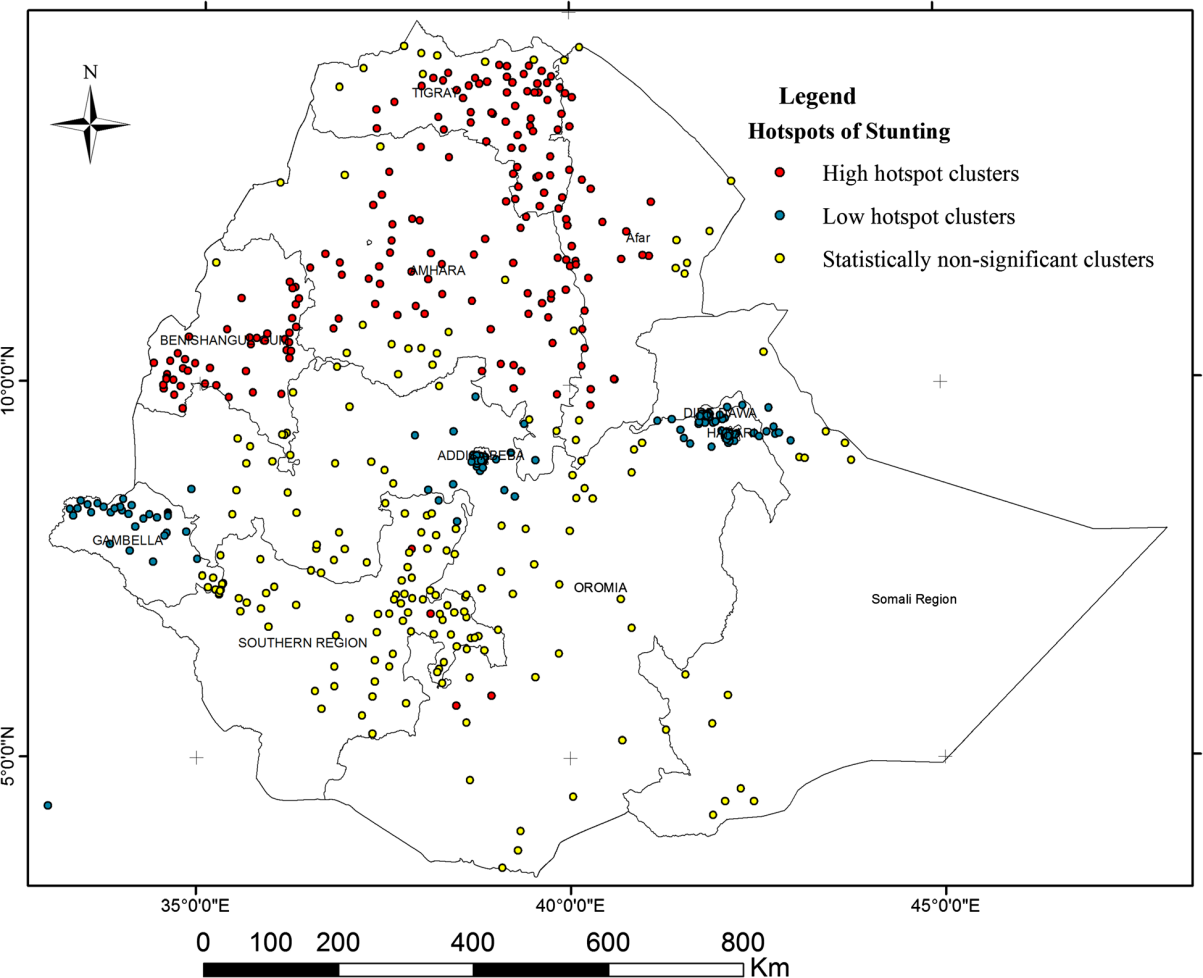
Statistical significant hotspots of childhood stunting at cluster level, DHS 2011

Figure 2 shows the spatial variation of stunting at the cluster level (lower level). The spatial analysis at the cluster level shows that statistically significant high hotspots of stunting were found in northern parts of the country(Amhara, Benishangul-Gumuz, Tigray and Affar regions), whereas statistically significant low spots of stunting were found in the western (Gambella), central (Addis Ababa) and eastern (DireDawa) parts of the country (Table 4 and Fig. 2).

**Table 4:** Hotspot and coldspot analysis of stunting among enumeration areas (clusters) per regional state in Ethiopia, EDHS 2011

Region	Total number of clusters	High hotspots*	Low hotspots**	Non-significant clusters***
Tigray	58	52	1	5
Afar	42	28	2	12
Amhara	68	40	4	14
Oromiya	73	3	21	49
Somali	35	-	16	19
Benishangul-Gumuz	46	39	1	6
SNNP	79	1	3	75
Gambela	46	1	33	12
Harari	42	-	39	3
Addis Ababa	52	-	49	3
Dire Dawa	40	-	39	1

*GiZScore positive (1.968–4.68) and *p*-value <0.05

**GiZScore negative (−6.42 to −2.623) and *p*-value <0.05

***GiZScore either positive or negative and *p*-value >=005

### Multilevel analysis

The results of multilevel logistic regression models for individual and community level factors are displayed in Table 5. In the final full model where all individual and community level factors are included, child’s age; gender; birth interval; maternal body mass index (BMI); education status of mother; educational status of father; sex of the household head; child’s anemia status; low household wealth index; region; and lack of availability of an improved latrine were factors that were significantly associated with childhood stunting.

**Table 5:** Factors associated with childhood stunting in Ethiopia by multilevel logistic regression analysis, EDHS 2011

Variables	Model 2	Model 3	Model 4
	AOR(95 % CI)	AOR(95 % CI)	AOR(95 % CI)
Individual level factors			
Child factors			
Child’s age(months)			
0–11	1.00		1.00
12–23	4.13(3.23–5.27)		4.12(3.22–5.26)
24–35	6.47(5.07–8.25)		6.61(5.17–8.44)
36–47	6.37(5.00–8.10)		6.44(5.10–8.20)
48–59	5.58(4.38–7.12)		5.65(4.43–7.21)
Sex			
Female	1.00		1.00
Male	1.20(1.08–1.37)		1.20(1.08–1.33)
Immunization			
Complete	1.00		1.00
Incomplete	1.01(0.76–1.34)		1.01(0.76–1.34)
Anemia			
Non-anemic	1.00		1.00
Mild	1.36(1.18–1.56)		1.43(1.24–1.64)
Moderate	1.64(1.42–1.89)		1.76(1.52–2.03)
Severe	2.93(2.14–4.01)		3.23(2.35–4.43)
Maternal/household factors			
Mother’s age (years)			
15–24	1.00		1.00
25–34	0.95(0.82–1.10)		0.96(0.83–1.11)
35–49	0.92(0.76–1.10)		0.93(0.77–1.12)
Maternal education			
No education	1.00		1.00
Primary	0.88(0.76–1.02)		0.90(0.78–1.04)
Secondary	0.64(0.41–1.01)		0.70(0.44–1.10)
Higher	0.43(0.19–0.97)		0.42(0.18–0.94)
Mother’s BMI (kg/m^2^)			
18.5–24.9	1.00		1.00
<18.5	1.08(0.95–1.22)		1.13(1.00–1.28)
> = 25.0	0.65(0.49–0.85)		0.69(0.52–0.90)
Birth interval			
<24 months	1.00		1.00
> = 24 months	1.72(1.50–1.98)		1.68(1.46–1.93)
Number of under five children			
One	1.00		1.00
Two	0.88(0.77–1.01)		0.90(0.79–1.03)
Three	0.88(0.73–1.06)		0.95(0.79–1.14)
Four	0.79(0.58–1.19)		0.90(0.61–1.33)
Head of household			
Female	1.00		1.00
Male	1.14(0.98–1.32)		1.18(1.01–1.38)
Father’s education level			
No education	1.00		1.00
Primary	0.98(0.87–1.11)		0.98(0.87–1.12)
Secondary	0.64(0.49–0.85)		0.74(0.56–0.99)
Higher	0.50(0.33–0.76)		0.58(0.38–0.89)
Family size			
≤4	1.00		1.00
5–7	1.03(0.88–1.20)		1.03(0.88–1.20)
8–10	1.05(0.86–1.27)		1.05(0.86–1.28)
≥11	1.21(0.86–1.71)		1.20(0.84–1.70)
Wealth index			
Richest	1.00		1.00
Richer	1.74(1.39–2.17)		1.46(1.12–1.90)
Middle	1.57(1.25–1.98)		1.24(0.94–1.64)
Poorer	1.82(1.45–2.29)		1.42(1.08–1.88)
Poorest	1.73(1.39–2.17)		1.43(1.08–1.89)
Community level factors			
Place of residence			
Urban		1.00	1.00
Rural		1.79(1.49–2.16)	1.18(0.88–1.60)
Poverty rate			
Low		1.00	1.00
High		1.02(0.88–1.20)	1.01(0.84–1.23)
Region			
Dire Dawa		1.00	1.00
Tigray		1.63(1.23–2.16)	1.58(1.09–2.29)
Affar		1.65(1.23–2.21)	1.27(0.86–1.86)
Amhara		1.62(1.23–2.15)	1.50(1.04–2.17)
Oromiya		1.01(0.77–1.32)	1.13(0.79–1.62)
Somali		0.80(0.59–1.09)	0.68(0.46–1.01)
Benishangul-Gumuz		1.49(1.11–2.01)	1.71(1.16–2.52)
SNNP		1.17(0.89–1.54)	1.43(1.00–2.06)
Gambela		0.57(0.47–0.78)	0.57(0.37–0.86)
Harari		0.73(0.52–1.01)	0.72(0.46–1.12)
Addis Ababa		0.78(0.53–1.14)	1.03(0.57–1.83)
Improved latrine facility			
Yes		1.00	1.00
No		1.27(1.07–1.51)	1.26(1.01–1.59)
Drinking water supply			
Piped water		1.00	1.00
Other improved		1.13(0.97–1.32)	1.03(0.85–1.25)
Unimproved		1.06(0.87–1.29)	1.04(0.87–1.24)

Model 1 is empty model, 1.00 = reference

### Individual level factors

Children 24–35 months old were 6.61 times (AOR = 6.61; 95 % CI: 5.17–8.44) more likely to be stunted than children less than one year old. The odds of stunting were increased by 20 % (AOR = 1.20; 95 % CI: 1.08–1.33) in male children compared to females. The odds of stunting among children with severe anemia were 3.32 times higher (AOR = 3.23; 95 % CI: 2.35–4.43) than in children with no anemia. Children with mothers who completed higher education were 58 % (AOR = 0.42; 95 % CI: 0.18–0.94) less likely to be stunted compared to those children whose mothers had no formal education. The odds of stunting were42% lower among children with fathers who completed higher education (AOR = 0.58; 95 % CI: 0.38–0.89) compared to children whose fathers had no formal education.

Children with mothers who had a high BMI (≥25.0 kg/m^2^) (AOR = 0.69; 95 % CI: 0.52–0.90) were less likely to be stunted compared with children whose mothers had normal BMI (18.5 kg/m^2^-24.9 kg/m^2^). The odds of being stunting were 68 % higher among children with a shorter (<24 months) birth interval compared to children with longer birth interval (≥24 months) (AOR = 1.68; 95 % CI: 1.46–1.93). Children from the poorest wealth quintile had 43 % higher odds of being stunted compared with children from the richest wealth quintile (AOR = 1.43; 95 % CI: 1.08–1.88). Children from male-headed households were 18 % more likely to be stunted compared to children from female-headed households (AOR = 1.18; 95 % CI: 1.01–1.38).

### Community level factors

Children from households without access to an improved latrine (defined as private and hygienic with a cleanable slab) had 26 % higher odds of stunting compared to children from households that reported access to an improved latrine(AOR = 1.26; 95 % CI:1.01–1.59). The odds of childhood stunting in Tigray (AOR =1.58; 95 % CI: 1.09–2.29), Amhara (AOR 1.50; 95 % CI 1.04–2.17) and Benishangul-Gumuz(AOR =1.71; 95 % CI: 1.16–2.52) were higher compared to Dire Dawa respectively. However the odds of childhood stunting at Gambella region were lower by 43 % as compared to Dire Dawa (AOR = 0.57; 95 % CI: 0.37–0.86) (Table 5).

As shown in Table 6, the empty model (the null model) revealed that childhood stunting was not random across the communities (τ^2^ = 0.363, *P <* 0.001). About 9.9 % of the variance in the odds of childhood stunting could be attributed to the community level factors as calculated by the ICC based on estimated intercept component variance. The full model, after adjusting for individual and community level factors, shows that the variation in childhood stunting across communities remained statistically significant. About 36.6 % of the odds of childhood stunting variation across communities was observed in the full model.

**Table 6:** Results from random intercept model (measure of variation) for childhood stunting at cluster level by multilevel logistic regression analysis

Measure of variation	Model 1^a^	P-value	Model 2^b^	p-value	Model 3^c^	p-value	Model 4^d^	p-value
Community level								
Variance (SE)	0.363(0.043)	<0.001	0.323(0.050)	<0.001	0.159(0.046)	<0.001	0.230(0.041)	<0.01
Explained variation(PCV)	Reference		11.0		56.2		36.6	
ICC (%)	9.9		8.9		4.6		6.5	
MOR	2.10		1.93		1.59		1.75	
Model fit statistics								
DIC (−2log likelihood)	13256		8499		12733		8357	

*SE* standard error, *ICC* intracluster correlation, *MOR* median odds ratio, *DIC* deviation information criterion

^a^Model 1 is the empty model, a baseline model without any determinant variable

^b^Model 2 is adjusted for individual-level factors

^c^Model 3 is adjusted for community-level factors

^d^Model 4 is final model adjusted for both individual and community-level factors

Moreover, the MOR confirmed that childhood stunting was attributed to community level factors. The MOR for stunting was 2.01 in the empty model; this indicated that there is variation between communities (clustering) since MOR is two times higher than the reference (MOR = 1). The unexplained community variation in stunting decreased to MOR of 1.75 when all factors were added to the null model (empty model). This shows that when all factors are considered, the effect of clustering is still statistically significant in the full model.

## Discussion

This study found that the prevalence of stunting was above the national average of 44 % in six out of the eleven regions of the country. Excess cases of stunting were found in the northern parts of the country characterized by highlands and midlands. This finding is consistent with the study done by Hagos et al. which found that a higher prevalence of stunting was found in the highlands and midlands compared to the lowlands [24]. That study concluded that rainfall and temperature are the main predictors of stunting variation across the country. Other studies also showed that climate change could indeed increase stunting rates in areas of the country dependent on rain-fed agriculture [25, 26]. The spatial variation of childhood stunting is also determined by environmental or geographical factors (e.g. population density, climate and disease environment) in addition to the individual and household level factors [27]. In this study Getis-Ord spatial statistics showed spatial variation of childhood stunting at the cluster level. Statistically significant hotspot areas of childhood stunting were found particularly in the northern parts of the country, in Benishangul-Gumuz, Amhara, Tigray and Affar regions, when we compare to clusters found in other regions. Exploring spatial variation is important to identify aggregations of cases in order to target nutritional interventions [8]. The identified clusters might be the areas where childhood stunting prevention and control interventions should be given priority [28].

This study confirmed that the variation in childhood stunting can be attributed to both individual and community level factors. In the final model, individual and community-level factors accounted for about 36.6 % of the variation observed for childhood stunting. The national average for stunting in children under five years of age in Ethiopia is 44.4 %. Children aged >12 months were at higher odds of being stunted compared to infants of less than one year. The trend of stunting increased for each age group of children age up to 35 months, and then declined to just under 50 % for children aged 48–59 months. This finding is consistent with many previous studies [29–34]. These studies reported that there is a rapid fall in children’s height-for-age Z-score from birth to 24 months, particularly during the time when children are being weaned off of exclusive breastfeeding and also becoming more mobile and crawling. These activities expose the child to contaminants in water and food, as well as soil and contaminants picked up on their hands that then go into their mouths. There continues to be an increase in stunting after 24 months, but the rate of increase is much slower. The cumulative effect in older age children might be one possible justification for this pattern. Studies report that children living in most developing countries are introduced directly to the regular household diet made of cereal or starchy root crops, which is the major cause for the high incidence of child malnutrition and morbidity [35–37]. Another article found that linear growth failure occurs within a complex interplay of more distal community and societal factors, such as access to healthcare and education, political stability, urbanisation, population density and social support networks [38].

This study found that male children had a slightly higher chance of being stunted than females. This result is consistent with previous studies [31, 39–41]. One possible explanation for this could be that childhood morbidity is higher among males than females in early life, even after adjusting for gestational age and body size [42]. Moreover, the proportion of male preterm births is higher than female preterm births which could also contribute to childhood stunting [39, 42–44]. However there are also studies which showed that stunting was not significantly associated with sex [32, 45], and programmatically this difference in stunting rates may not be useful for targeting interventions.

Maternal education was found to be significantly negatively associated with childhood stunting. Many previous studies have found that maternal education has a positive effect on reducing childhood stunting [29, 31, 46–51]. The knowledge that mothers acquire from formal education could help them to adopt essential nutrition and hygiene behaviors that prevent childhood stunting. Another possible reason might be that educated mothers have better health-seeking behavior for childhood illnesses as compared to uneducated mothers which can help prevent stunting [52, 53]. Besides maternal education, father’s education was also significantly associated with childhood stunting. Those children whose fathers had attended formal education had less chance of being stunted. Similar findings have been reported by other studies [46, 54]. Those fathers with formal education might be more knowledgeable on proper child feeding and hygiene practices, which have a positive effect on preventing childhood stunting. Father’s higher education has also been associated with good health seeking behavior for childhood illness [55]. In Ethiopia lack of education is a widespread problem, about 59 % and 33 % of the women and men were illiterate, respectively which shows how this factor is important in prevention of stunting in Ethiopia [10].

The socioeconomic status of households is associated with access to nutritious foods at the household level, which in turn determines the growth and development of children [56]. Findings of this study revealed that children from households in the poorest wealth quintile were more likely to be stunted than children from the richest quintile, which is consistent with the findings of previous studies carried out in different developing countries [29, 46–49, 57–59]. This could be due to the fact that increased income improves dietary diversity [60, 61], which in turn improves the adequacy of nutrient intake and nutritional status, and underscores the importance of linking income generating activities with other nutritional interventions. In Ethiopia, above 45 % of the population was found in poor and poorest wealth quintile, which shows the proportion of children who are at risk for stunting due to low socioeconomic status [10].

The 2014 Mini EDHS data show that only 9.7 % of the population had access to an improved and shared latrine facilities [62]. This study found that the availability of an improved latrine was one of the significant factors associated with childhood stunting, which is consistent with other studies conducted in developing countries [41, 63]. One explanation could be a reduction in the pathogen load in the environment from correct and consistent use of improved sanitation, since exposure to feces has been linked to environmental enteropathy and stunting [64–66].

A short birth interval is common in Ethiopia and a known risk factor for stunting [67, 68]. Twenty percent of the births have an interval of less than two years, and 9 percent of births are less than 18 months apart. This implies that 20 % of the Ethiopian children were at risk of stunting attributable to short birth interval [10]. This study found that having a birth interval ≥ 24 months decreased the odds of being stunted. This is consistent with other studies [68, 69].A short interval between births can have an adverse effect on child nutrition by causing intrauterine growth retardation, and/or undermining the quality of child care [70].

Birth spacing might also influence childhood under-nutrition through its association with preterm births and low birth weight. If a pregnancy occurs too soon after the previous birth, the mother may not recover her nutritional status, which can contribute to preterm birth and low birth weight [68]. Maternal nutritional status was associated with childhood stunting in this study. The prevalence of under nutrition (BMI < 18.5 kg/m) among women in Ethiopia was 27 % [10]. Mothers who had a BMI <18.5 kg/m^2^ were marginally associated with higher odds of childhood stunting compared to mothers with normal BMI (18.5 kg/m^2^ -24.9 kg/m^2^). Mothers who had BMI ≥25 kg/m^2^wereless likely to have child who is stunted. A study from Brazil also suggested that maternal nutritional status was associated with child nutritional status [71]. A study from Cambodia also found that maternal BMI was associated with childhood stunting [69].

The representativeness of the data with a large sample size, as well as a nationally representative population-based study with a high response rate are strengths of this study that give high statistical power to infer the characteristics of the study population. Another important strength of this study is the use of multilevel logistic regression analysis, which was able to identify other factors beyond individual-level factors that would not be identified by using standard logistic regression analysis. The use of a combination of methods (spatial and regression statistics) was a strength which allowed validation of the identified hotspots areas due to the assumptions of the statistical methods. One limitation is that the cross-sectional nature of the study means the results cannot be used to establish cause and effect relationship, and is a limitation of the study. Another limitation is the absence of other important variables like behavioral factors and quantitative dietary consumption to substantiate the findings, since the EDHS survey was designed to produce health and health related indices, but was not specifically administered for the purposes of this study.

## Conclusion

This study found that childhood stunting was not random in Ethiopia. Statistically significant high hotspots of stunting were found in northern parts of the country whereas low hotspots of stunting were found in central, eastern, western parts of the country. Individual and community level factors accounted for 36.6 % of the variation in childhood stunting across the communities. Both individual- and community-level factors were significant determinants of childhood stunting. Being male, age above 11 months, short birth interval, having anemia, no formal education of mother and/or father, being from male-headed household, and being from a household in the lowest wealth quintile were the factors that increased the odds of stunting at the individual level, whereas a lack of access to an improved latrine and residence in the northern regions of the country were the factors associated with stunting at the community level.

Thus, the improvement of nutritional status of children requires multi-factorial interventions such as reducing poverty and ensuring food security, ensuring adequate birth interval, educating mothers and their partners, a healthy environment and good hygienic practices enabled by access to hygienic latrines. The regions identified as having high hotspots of childhood stunting should be prioritized for nutritional interventions.

## Abbreviations

AOR: adjusted odds ratio
CI: confidence intervals
EDHS: Ethiopian demographic health survey
GIS: geographic information system
HAZ: height-for-age
ICC: intra-cluster correlation
MOR: median odds ratio
PCV: proportional change in variance
SD: standard deviation
WHO: world health organization
